# Identification of a Secreted Casein Kinase 1 in *Leishmania donovani*: Effect of Protein over Expression on Parasite Growth and Virulence

**DOI:** 10.1371/journal.pone.0079287

**Published:** 2013-11-15

**Authors:** Mary Dan-Goor, Abedelmajeed Nasereddin, Hanan Jaber, Charles L. Jaffe

**Affiliations:** Department of Microbiology and Molecular Genetics, The Kuvin Center for the Study of Infectious and Tropical Diseases, National Center for Leishmaniasis, IMRIC, Hebrew University–Hadassah Medical School, Jerusalem, Israel; Institut national de la santé et de la recherche médicale - Institut Cochin, France

## Abstract

Casein kinase 1 (CK1) plays an important role in eukaryotic signaling pathways, and their substrates include key regulatory proteins involved in cell differentiation, proliferation and chromosome segregation. The *Leishmania* genome encodes six potential CK1 isoforms, of which five have orthologs in other trypanosomatidae. *Leishmania donovani* CK1 isoform 4 (*Ldck1.4*, orthologous to LmjF27.1780) is unique to *Leishmania* and contains a putative secretion signal peptide. The full-length gene and three shorter constructs were cloned and expressed in *E. coli* as His-tag proteins. Only the full-length 62.3 kDa protein showed protein kinase activity indicating that the N-terminal and C-terminal domains are essential for protein activity. LdCK1.4-FLAG was stably over expressed in *L. donovani*, and shown by immunofluorescence to be localized primarily in the cytosol. Western blotting using anti-FLAG and anti-CK1.4 antibodies showed that this CK1 isoform is expressed and secreted by promastigotes. Over expression of LdCK1.4 had a significant effect on promastigote growth in culture with these parasites growing to higher cell densities than the control parasites (wild-type or Ld:luciferase, P<0.001). Analysis by flow cytometry showed a higher percentage, ∼4–5-fold, of virulent metacyclic promastigotes on day 3 among the LdCK1.4 parasites. Finally, parasites over expressing LdCK1.4 gave significantly higher infections of mouse peritoneal macrophages compared to wild-type parasites, 28.6% versus 6.3%, respectively (p = 0.0005). These results suggest that LdCK1.4 plays an important role in parasite survival and virulence. Further studies are needed to validate CK1.4 as a therapeutic target in *Leishmania*.

## Introduction

*Leishmania* are protozoan parasites responsible for a variety of human diseases ranging from simple self-healing cutaneous leishmaniasis to the fatal visceral form of the disease. According to the World Health Organization more than 12 million people in ∼88 countries have leishmaniasis, with approximately 1–2 million new cases annually adding to the existing global burden of disease. Most of the countries where these diseases are endemic are developing countries, and these diseases are frequently associated with poverty, malnutrition and environmental changes [1], [2].

*Leishmania* have a digenetic life-cycle existing as extracellular flagellated promastigotes in sand fly vectors; and as intracellular aflagellated amastigotes in the macrophages and dendritic cells of their mammalian hosts [3], [4]. In eukaryotic cells protein phosphorylation is a major mechanism of signal transduction, and involved in regulation of many different cellular processes including differentiation, cell division, and host-pathogen responses [5]–[7]. Previously it was demonstrated that protein kinases, including casein kinase 1 (CK1) and casein kinase 2 (CK2), are released/secreted by promastigotes of several *Leishmania* species [8]–[13]. Constitutive or induced release of CK1 and CK2 from promastigotes could be modulated by temperature and pH [11], two important environmental cues for leishmanial differentiation from promastigotes to amastigotes and visa versa. Modification of temperature (34–37°C) and acidic pH, have been utilized for axenic amastigote propagation *in vitro* and studies on parasite differentiation [14], [15].

Enzymes belonging to the CK1 family are found in all eukaryotes from protozoa to humans where they phosphorylate a wide range of protein substrates involved in various processes including: cell cycle, receptor signaling, transport, apoptosis, transcription, and DNA repair [16], [17]. Several CK1 isoforms have been characterized in yeast, and at least six isoforms have been identified in humans [16]. The catalytic domains of CK1 isoforms are highly conserved, but the N-terminal and C-terminal non-catalytic domains differ significantly in both length and primary amino acid sequence [16], [17]. CK1 isoforms tend to be constitutively expressed, and characterized by an acidophilic target phosphorylation sites. These sites are frequently adjacent serine/threonine residues phosphorylated by other protein kinases allowing CK1 to act in hierarchical manner and further modulated activity of other protein kinases [16], [17].

Analysis of the TriTryp kinome identified multiple CK1 isoforms in *L. major* (six), *Trypanosoma cruzi* (seven) and *T. brucei* (four) [18], of which four are conserved among trypanosomatids [18], [19]. CK1 isoform two (CK1.2), present in all three trypanosomatids, appears to be essential for parasite growth. Knockdown of *ck*1.2 (Tb927.5.800) expression in *T. brucei* bloodstream forms results in major morphological changes and death [20], while protein kinase inhibitors inhibiting *Leishmania* promastigote growth were shown to bind and inhibit leishmanial CK1.2 (LmjF35.1010) [21], [22].

CK1 isoforms in other organisms have been localized to specific subcellular environments including the nucleus, cytosol, and plasma membrane [16]. Recently analysis of the *Leishmania* secretome using conditioned culture medium showed that CK1.2 is released by promastigotes, and appears to be associated with exosomes released by the parasites [8], [12]. Expression of *L. major* CK1.2 in mammalian cells stimulated the phosphorylation-dependent degradation of the IFNAR1 chain of the IFN type I receptor and attenuation of IFN-α/β signaling [23], suggesting that secreted CK1s may also play a role as parasite virulence factors.

Further analysis of the TriTryp kinome identified a CK1 isoform (LmjF27.1780, isoform 4) unique to *Leishmania* that appears to have a secretion signal sequence [18]. Here we report on the cloning, expression and molecular characterization of CK1 isoform 4 (CK1.4) from *L. donovani*. Over expression in *L. donovani* demonstrates that isoform 4 is secreted by the parasite, and plays a role in parasite growth and survival. These results encourage further investigations of leishmanial CK1.4 as a potential chemotherapeutic target.

## Materials and Methods

### 1. Animals

All procedures used for animal experiments were approved by the Hebrew University Animal Studies Ethical Committee.

### 2. Cell Culture

*Leishmania donovani* (MHOM/SD/1962/1S-Cl 2d) wild-type promastigotes (Ld:wt) were grown in M199 medium containing 10% fetal calf serum and antibiotics at 26°C as previously described [24].

### 3. Cloning and Sequencing of *L. donovani* ck1 Isoform 4 (*Ldck1.4*)

*Ldck1.4* was amplified in a T Gradient PCR machine (Biometra, Goettingen, Germany) from *L. donovani* genomic DNA using the oligonucleotide primers: MFCK1∶5′- CCC CGG ATC CAT GAC GCT GAC GAG CCG TAC C -3′ and MRCK1∶5′- CCC CGA GCT CTT AAC GCA TCT GCC GCA GCT G -3′). The reaction mixture contained: 50 µM each primer, 1.25 Unit Platinum *Pfx* DNA polymerase (Invitrogen, Carlsbad, CA), 1 mM MgCl_2_, 0.4 mM dNTPs, and 1x Platinum *Pfx* DNA polymerase buffer (final volume 50 µl), and was carried out using the following conditions: initial denaturation at 98°C for 3 min followed by 35 cycles of denaturation at 95°C, 15 sec; annealing at 55°C, 30 sec; and extension at 72°C, 60 sec. Final extension was carried out for 6 min at 72°C. The 1684 bp PCR product was analyzed by electrophoresis in 1.2% agarose gels containing 0.25 µg/µl ethidium bromide and purified using Wizard SV Gel and PCR Clean-Up System (Promega, Madison, WI). After cutting with Fastdigest *Bam*HI (2 µl) and *Sac*I (2 µl) for 2 hrs, at 37°C the product was cloned into pET28a, the insert sequenced (Center from Genomics, Hebrew University of Jerusalem) and submitted to NCBI GeneBank (Accession No. JN225463). Predicted protein sequence alignment and motif analysis was carried out using ClustalW2 (http://www.ebi.ac.uk/Tools/msa/clustalw2/) and motif scan (http://myhits.isb-sib.ch/cgi-bin/motif_scan), respectively. Blast analysis using both the full-length protein and the conserved protein kinase catalytic domain was carried out (http://blast.ncbi.nlm.nih.gov/Blast.cgi). Phylogenetic and the molecular evolutionary relationship of the conserved CK1 catalytic domains from different organism was analyzed using MEGA version 5.2 [25]. The TritrypDB Version 3.2 [26] (http://tritrypdb.org/tritrypdb/) was used to identify orthologs of putative leishmanial casein kinases in other Kinetoplastid parasites. Analysis of classical and non-classical peptide secretion via mammalian, gram-negative and gram-positive secretion pathways was carried out using SignalP version 3.0 (http://www.cbs.dtu.dk/services/SignalP) and SecretomeP version 2.0 (http://www.cbs.dtu.dk/services/SecretomeP), respectively [27].

### 4. Expression of Full-length Recombinant LdCK1.4 and Deletion Constructs in *E. coli*

Full-length *ck1.4* was amplified from *L. donovani* genomic DNA as described in section 3. In addition, three deletion constructs were prepared by PCR: *ck1.4* minus nucleotides 1–270 (ck1.4Δ5′), *ck1.4* minus nucleotides 1231–1707 (*ck1.4Δ3′*), and *ck1.4Δ5′Δ3′*. The following primers were used to prepare the deletion constructs: *ck1.4Δ5′* (PFCK1, 5′- CCC CGG ATC CCA GCA GGG CCG CAG TAA CCA -3′ and MRCK1); *ck1.4Δ3′* (MFCK1 and P2MRCK1, 5′- GGG GGA GCT CCT GGT ACT GTG GCT CCT CTT C -3′); *ck1.4Δ5′Δ3′* (PFCK1 and P2MRCK1).

PCRs were carried out using the same conditions described for the full-length construct in section 3. The amplification products (*ck1.4* - 1707 bp, *ck1.4Δ5′* - 1437 bp, *ck1.4Δ3′* - 1230 bp, and *ck1.4Δ5′Δ3′* - 960 bp) were analyzed in 1.2% agarose gels, cloned directly into pET28a, and used to transform competent BL21-A1 bacteria.

Optimal expression of soluble recombinant polypeptides was achieved at 22°C in BL21-A1 bacteria by adding L-arabinose (0.2%) and IPTG (1 mM) for 18 hrs. Cell pellets were suspended in lysis buffer (20 mM Tris pH 7.4, 150 mM NaCl, 50 mM NaF, 1 mM EDTA, 0.2 mM Brij, 0.1 mM PMSF and 0.07% β-mercaptoethanol) containing 1 mg/L lysozome. After sonication on ice (3 min, Transsonic T310 bath, Elm GmbH, Germany) the supernatants were collected by centrifugation (10000×g for 30 min) and analyzed by SDS-PAGE on 12% gels by Coomasie Blue staining or Western blotting. After transferring to nitrocellulose membranes the recombinant proteins were detected with Nickel conjugated Horseradish peroxidase (1/10000 dilution, HisDetector Nickel-HRP, KPL Inc, USA) as described in section 6.

Purification of the full-length expression protein LdCK1.4 for rabbit polyclonal antibodies, section 6, was carried out essentially as described (Qiagen, 2003). In brief, bacteria were suspended in lysis buffer and sonicated on ice. The supernatant was collected by centrifugation and incubated with Ni-NTA agarose beads (300 µl, Qiagen GmbH, Germany) for 1 hr at 4°C. Imidazole (10 mM) was added and the beads incubated for an additional 30 min at room temperature. After rinsing 3 times with wash buffer (lysis buffer plus 10 mM imidazole), the bound material was eluted from the resin in three fractions using a step gradient containing 100 mM, 500 mM or 1000 mM imidazole in lysis buffer. Each fraction was analyzed for purity following SDS-PAGE on 12% gels and staining with either Coomasie Blue or western blotting.

### 5. Production of *L. donovani* Promastigotes Over Expressing Either CK1 Isoform 4 Tagged with the FLAG Epitope (Ld:CK1.4-FLAG) or Luciferase (Ld:LUC)

The full-length *ck1.4* gene was amplified from *L. donovani* genomic DNA (20 ng) using the primers Ck1I3F 5′- CAC CAT GAC GCT GAC GAG C -3′ and Ck1I3Rev 5′- ACG CAT CTG CCG CAG CT -3′, and the product gel purified using the Wizard SV Gel and PCR Clean-Up System (Promega, USA). This material was used as a template for a second PCR, primers Ck1I3F and CK1FlagR 5′- TTA CTT GTC GTC ATC GTC TTT GTA GTC ACG CAT CTG CCG CAG CT -3′, in order to introduce a DNA sequence coding for a FLAG-tag at the 3′- terminus. The PCR mixture for both reactions contained: 50 µM each Primer, 1.25 Unit Platinum *Pfx* DNA polymerase (Invitrogen, CA), 1 mM MgCl_2_, 0.4 mM dNTPs, and 1x Platinum *Pfx* DNA polymerase buffer (final volume 50 µl). PCR conditions were 95°C for 5 min, followed by 35 cycles at 95°C for 20 sec, annealing at 53°C for 30 sec, and elongation at 72°C for 2 min. The final elongation step was carried out at 72°C for 10 min. The amplicon was cloned into the pENTR/TEV/D-TOPO plasmid according to the manufacturer’s (Invitrogen, CA) instructions, and then used to transform One Shot chemically competent *E. coli*. Colonies containing an insert were selected on LB plates containing 50 µg/ml kanamycin, grown in LB medium overnight, and plasmid DNA purified (QIAprep Spin Miniprep Kit, Qiagen). Presence of full-length gene was checked by digestion with restriction enzymes (*EcoR*V/*Xho*I and *Pst*I). Transfer of *Ldck1.4-FLAG* by LR reaction into the leishmanial Gateway destination vector pSSU-int/RFB (see below) was carried out essentially as described by the manufacture for other destination vectors using Gateway cloning technology (Invitrogen, CA), except that the final incubation was for 2 hrs at 25°C. Positive colonies were examined for the presence of the *ck1.4* gene by PCR (Forward primer pSSUF 5′- ACACAAAAGGCGTGAAAAC and CK1FlagR) and the insert sequenced. The plasmid pSSU-int/RFB:ck1.4-FLAG (5 µg) was digested with *Pme*I and *Pac*I, purified, and the linearized vector used to transfect *L. donovani* promastigotes essentially as previously described [28]. Linearized DNA (5 µg) was added to 400 µl parasites (10^8^ cells) diluted in cytomix buffer in a EC Gene Pulser cuvette (2 mm cuvette, BioRad, Hercules, CA), and pulsed once with 1600 V, 200 OEM, 25 µF (ECM 630, BTX, Holliston, MA). Parasites were incubated on ice for 10 min, and cultured for 24 hrs after which stably transfected Ld:CK1.4-FLAG (mutant) promastigotes were selected with hygromycin (25 µg/ml).

Parasites stably expressing luciferase *Ld*:pSSU-int/LUC (Ld:LUC) were prepared essentially as described [29], except that *L. donovani* promastigotes were transfected with the linearized plasmid pSSU-int/LUC. Mutant parasites selected with hygromycin (25 µg/ml).

The leishmanial Gateway destination vector pSSU-int/RFB was prepared as follows: Reading Frame Cassette B (RfB, Invitrogen) was cloned into pBluescript using the EcoRV restriction site, digested with *Cla*I and *Spe*I, and then cloned into the polylinker of the predigested plasmid pSSU-int/β-GAL [30] which was a gift from T. Aebischer, Robert Koch Institute, Germany. The destination vector was used to transform DB3.1 cells, and plasmid DNA purified from mini-preparations of positive colonies (QIAprep Spin Miniprep Kit, Qiagen).

### 6. Western Blotting

Expression of full-length and truncated recombinant CK1.4 by *E. coli,* as well as the full-length protein by Ld:CK1.4-FLAG and Ld:wt parasites, were examined by Western blotting. Proteins were separated by SDS-PAGE on acrylamide gels and transferred to nitrocellulose membranes. The membranes were blocked with 1% BSA, and incubated with either Nickel conjugated Horseradish peroxidase (1/10000 dilution, HisDetector Nickel-HRP, KPL Inc, USA), mouse anti-FLAG antibody (1/1000 dilution M2 monoclonal antibody, Sigma –Aldrich Chemical Co, USA), rabbit anti-CK1.4 antibody (1/7500 dilution), rabbit anti-KMP 11 antibody (1/10000 dilution) or rabbit anti-HSP83 (1/5000 dilution). After washing twice with 0.1% Tween in 20 mM Tris-buffered saline pH 7.5 (washing buffer), binding of the primary antibodies was detected after incubating with Rabbit anti-mouse IgG HRP (1/20000 dilution) or Protein A conjugated HRP (1/12000 dilution). After further washes, binding was detected by incubating with chemiluminescent substrate and exposure to X-ray film. Densiometric analysis was carried out using the NIH Image program (developed at the U.S. National Institutes of Health and available on the Internet at http://rsb.info.hih.gov/hih-image).

Polyclonal serum to CK1.4 and to KMP-11 was produced in New Zealand white rabbits immunized with purified recombinant protein in Freunds adjuvant; and antiserum to HSP83 was a generous gift of D. Zilberstein (Technion Israel Institute of Technology, Israel).

### 7. Immunofluorescence

Logarithmic *Leishmania* promastigotes (Ld:CK1.4 or Ld:LUC) were washed 3 times with phosphate buffered saline, pH 7.4 (PBS), and fixed with 4% (w/v) paraformaldehyde in PBS (20 min, room temperature). After fixation the parasites were washed with PBS, and suspended at 2×10^7^ promastigotes/ml. Parasites (200 µl/well) were added to 24-well microtiter plates containing poly-L-lysine coated glass cover slips and centrifuged for 20 min. Excess PBS was discarded, and the parasites permeabilized with cold methanol (7 min, −20°C). After rinsing with PBS the cells were blocked with 5% BSA in PBS (1 hr at room temperature), and then incubated (1 hr, room temperature) either with anti-FLAG M2 mouse monoclonal antibody (Sigma-Aldrich, 1/500 dilution), rabbit anti-CK1.4 antibody (1/7500 dilution) or buffer alone. The slides were washed and incubated for 30 min at room temperature with either Cy2-goat anti-mouse IgG or Cy3-goat anti-rabbit IgG (Jackson ImmunoResearch Laboratories, USA, 1/200 dilution), washed 3 times with PBS, mounted using Fluoroshield with DAPI (Sigma-Aldrich, USA), and examined by fluorescent microscopy. Images were taken using Apochromat oil immersion objective (100×magnification) on an Olympus IX71S8F microscope (Tokyo, Japan) equipped with Exi BlueTM Fast camera (QImaging, BC Canada).

### 8. Phosphorylation Assays

Protein kinase activity of *E. coli* lysates expressing full-length or different truncated recombinant LdCK1.4 was examined as previously described with the following modifications [10]. Bacterial lysates were prepared from cultures collected at the same cell density (OD _600 nm_ = 0.4) with and without induction, as described in section 4. Aliquots of the lysate (20 µl) were suspended in 40 µl of buffer A (20 mM Tris-HCl, pH = 7.5, 150 mM NaCl, 1 mM MgCl_2_, 1 mM glucose and 10 mM NaF) containing hydrolyzed casein (200 µg/ml), [γ-^32^P] ATP (1–10 µCi) and 0.1 mM cold ATP. Reactions were incubated for 10 min at 30°C and stopped by the addition of 85% phosphoric acid (5 µl). After 30 min on ice the samples (40 µl) were spotted on 2×2 cm squares of P81 phosphocellulose paper (Whatman), and washed four times with excess 0.5% phosphoric acid. The air-dried papers were placed in vials containing scintillation fluid, and counted in a β-scintillation counter (Kontron Liquid Scintillation Counter).

### 9. Analysis of CK1.4 Secretion by Ld:CK1.4-FLAG Mutants

Induced release of CK activity was carried out essentially as described [10] with the following modifications. Ld:CK1.4-FLAG or Ld:wt parasites (5×10^8^ promastigotes in buffer A containing 5 µM ionomycin and 1 mM EGTA at 28°C were layered over di-N-butyl phthalate, and cell-free supernatants (100 µl) collected at 0, 3, 5, 10, and 15 min. Western blot analysis was carried out as described above in section 6.

### 10. Mutant and Wild-type Promastigote Growth in Culture

Promastigotes, Ld:wt and Ld:CK1.4-FLAG, were diluted (5×10^5^ cells/ml) in complete culture media containing 10% Alamar blue solution (AbD Serotec, Oxford, OX5 1GE, UK) and aliquoted (250 µl/well) in sterile 96-well flat bottom plates. The plates were incubated at 26°C, and the fluorescence was read (λ_ex_ = 544 nm; λ_em_ = 590 nm) daily over 6 days using a fluorescent microplate reader (Fluoroskan Ascent FL, Finland). Promastigote (Ld:wt, Ld:CK1.4-FLAG and Ld:LUC) growth was also measured by counting live parasites daily in a Neubauer haemocytometer. All experiments were performed in triplicates. Results were analyzed using Prism 6 (GraphPad Software, San Diego, CA).

### 11. Analysis of Differentiation into Metacyclic Stage Promastigotes

Differentiation in culture of Ld:wt, Ld:CK1.4-FLAG and Ld:LUC parasites into metacyclic stage promastigotes over 7 days was determined by flow cytometry [31]. Samples were removed, washed and adjusted to 1.5×10^6^ cells/ml in ice cold PBS containing 10% FCS and 1% sodium azide. The cells were stained with propidium iodide (0.1 mg/ml) for 5 min, washed by centrifugation with FACS buffer (PBS containing 2% FCS and 0.01% sodium azide), and finally suspended in FACS buffer. Forward and side scatter parameters were collected with a flow cytometer (CyAn™ ADP Dako, Carpinteria CA, USA) and analyzed using Summitv4.3 software. Correct gating of procyclic and metacyclic promastigote populations (Figure S1) was determined by separation on Ficoll step gradients, as previously described [32], [33], and analysis by flow cytometry.

### 12. Infection of Mouse Macrophages by Mutant and Wild-type Promastigotes

Resident peritoneal macrophages were isolated from thioglycollate stimulated BALB/c mice [34] and allowed to adhere overnight (10^5^ cells/well, 37°C, CO_2_ incubator) to Lab-Tek II 8-well chamber slides (NUNC A/S, Denmark). Non-adherent cells were removed by washing with warm medium and the macrophages infected for 3 hrs (10/1 parasite/macrophage ratio) in quadruplicate with either Ld:wt, Ld:CK1.4-FLAG or Ld:LUC stationary phase promastigotes (day 5). Excess parasites were removed by washing 3 times with warm medium and the slides further incubated for 72 hrs. Slides were removed, stained with Diff-Quick (American Scientific Products, McGaw Park, IL), and the % infected macrophages and number of parasites per infected macrophage determined by light microscopy.

## Results

### 1. Cloning of Full-length *L. donovani CK1.4* Gene and Preparation of Deletion Constructs

**Figure 1:**
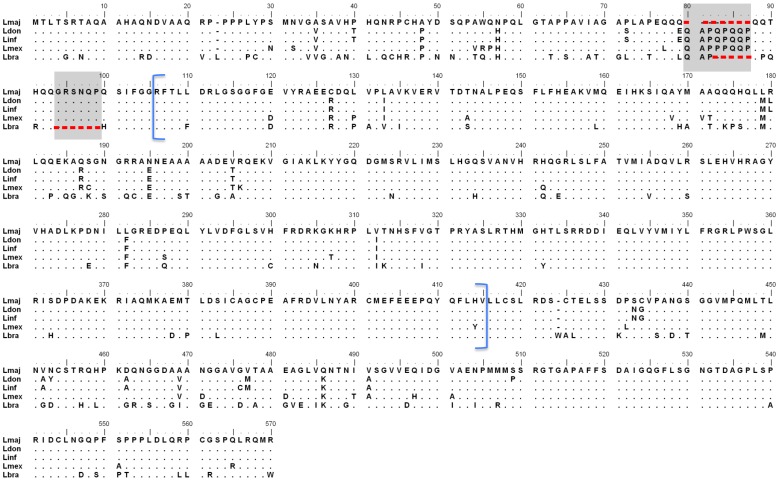
Multi-alignment of leishmanial CK1.4 protein sequences. Predicted protein sequence for CK1.4 orthologs from *Leishmania major* (Lmaj - Gene Bank Accession No. CBZ12245.1), *L. donovani* (Ldon - JN225463), *L. infantum* (Linf - CAM69136.1), *L. mexicana* (Lmex - CBZ28278.1) and *L. braziliensis* (Lbra - CAM45467.1). Identical amino acids are marked by dots, and the gaps by red dashes. The blue brackets delineate the conserved protein kinase domain.

DNA sequences for the *L. major* and *L. infantum ck1.4* orthologs (LmjF27.1780 and LinJ.27.1680, respectively) were aligned, and oligonucleotide primers (MFCK1 and MRCK1) to the conserved 5′- and 3′- regions of the gene used to amplify the gene from *L. donovani*. The DNA sequence obtained for *Ldck1.4* shows high homology to orthologs in other *Leishmania* species (99, 95, 95 and 82% homology over 568 amino acids for *L. infantum*, *L. major*, *L. mexicana* and *L. braziliensis*, respectively). The amino acid sequences for CK1.4 from *L. donovani* and *L. infantum* are almost identical (99% over 568 amino acids), likewise the *L. donovani* casein kinase shows high identity with the *L. major* and *L. mexicana* (LmxM27.1780) orthologs, 94% and 93%, respectively, over 568 amino acids (Figure 1). Interestingly, both the *L. major* and *L. braziliensis* (LbrM27.1900) orthologs have amino acid gaps (8 for *L. major*, and 5 and 6 for *L. braziliensis*; red dashes) in the NH_2_-terminal region of the enzyme just preceding the conserved catalytic region. *L. braziliensis* LdCK1.4 shows lowest identity to the *L. donovani* enzyme 77% over 569 amino acids. This perhaps is not surprising, as *L. braziliensis* and the other parasite species belong to different subgenera, *Viannia* and *Leishmania* respectively.

**Figure 2:**
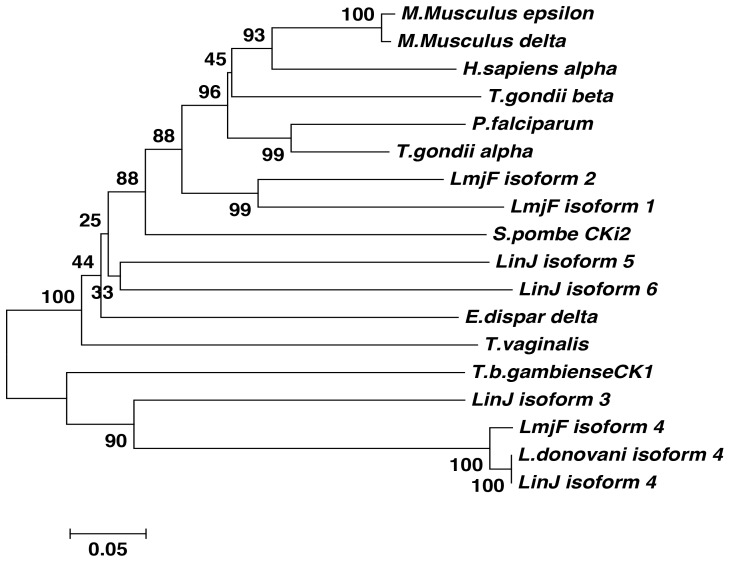
Dendrogram showing the evolutionary relationship of CK1 isoforms from different organisms. The tree was constructed by neighbor-joining analysis of the conserved catalytic domain from 18 CK1 sequences. Both complete and pairwise deletion options were tested and the bootstrap consensus tree built using 1000 replicates. Analyses were conducted using Mega (Molecular evolutionary genetic analysis) software, version 5.2 [25]. Sequences examined include: LmjF isoform 1 - *Leishmania major* (LmjF35.1000); LmjF isoform 2 - *L. major* (LmjF35.1010); LinJ isoform 3 - *L. infantum* (LinJ04.1230); *L. donovani* isoform 4 - (JN225463), LmjF isoform 4 - *L. major* (LmjF27.1780), LinJ isoform 4 - *L. infantum* (LinJ27.1680); LinJ isoform 5 - *L. infantum* (LinJ25.1640); LinJ isoform 6 - *L. infantum* (LinJ30.3530). Other organisms: *T. b. gambiense* CK1 (CBH15205.1), *Entamoeba dispar* δ isoform (XP_001735831.1), *Trichomonas vaginalis* (XP_001327672.1), *Plasmodium falciparum* (AAn35960), *Toxoplasma gondii α* isoform (XP_002366683), *T. gondii* β isoform (XP_00236367), *Schizosaccharomyces pombe* CKi2 (NP_595380), *Homo sapiens* α1 isoform (AAV_38633.1), *Mus Musculus* δ isoform (NP_082150.1), *Mus Musculus* ε isoform (NP_038795.3).

The protein kinase catalytic domain in LdCK1.4 is predicted to span amino acid residues 106 to 415 (Figure 1, blue brackets). Phylogenetic analysis (Figure 2) using only the conserved domain shows that isoforms 3 and 4, present on chromosomes 4 and 27 respectively, are most closely related (LdCK1.4 shows 46% identity and 60% homology over 310 amino acids to CK1.3, either LinJ04.1230 or LmjF04.1230), and form a separate clade from other leishmanial CK1 isoforms. CK1.4 is only found in *Leishmania* species, however isoform 3 does have orthologs in trypanosomes. Interestingly, the conserved protein kinase domain of isoforms 1 and 2 are more similar to mammalian casein kinases than to CK1.4. The conserved protein kinase domain of *L. infantum* CK1.2 shows 69% identity over 295 amino acids to *Mus musculus* CK1 epsilon (87% homology), but only 32% identity over 310 amino acids (50% homology) to LdCK1.4.

LdCK1.4 was analyzed using SecretomeP version 2.0 and SignalP version 3.0, programs that predict non-classical and classical protein secretion. The former program gives a SecP score = 0.7959 (normal threshold for secreted proteins >0.5), while the latter program predicts a short 13 amino acid signal peptide region with a protease cleavage site between amino acids 13 and 14.

### 2. Expression and Activity of Recombinant His-tag LdCK1.4 Polypeptides

**Figure 3:**
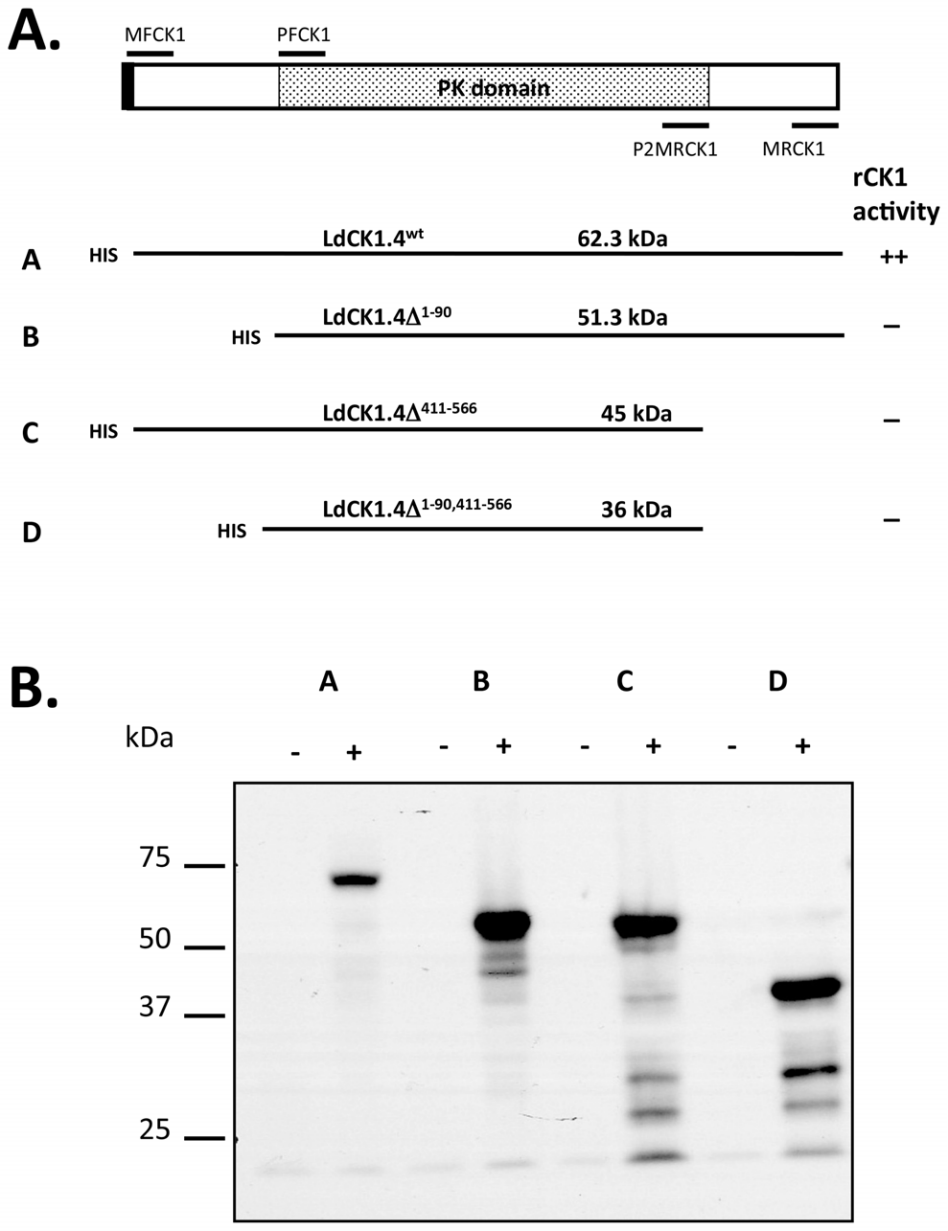
Expression of His-tag CK1.4 wild-type and deletion constructs. Panel A. Schematic description of recombinant full-length CK1.4 and truncated polypeptides. Conserved protein kinase catalytic domain – speckled box; predicted signal polypeptide – solid black box; (His)_6_ epitope tag – his; Oligonucleotide primers used for PCR: forward primers -MFCK1 and PFCK1; reverse primers – MRCK1 and P2MRCK1. Predicted molecular mass (kDa) of each recombinant protein (A – D) and casein kinase activity (rCK1) is given. Panel B. Western blot analysis of full length CK1.4 and truncated polypeptides expressed in *E. coli.* Expression of recombinant proteins was induced by addition of L-arabinose and IPTG for 18 hrs at 22°C (Lanes +). Non-induced bacteria were used as negative controls (Lanes −). Bacterial lysates were separated by SDS-PAGE, transferred to nitrocellulose membranes and probed with Nickel conjugated Horseradish peroxidase (1/10000 dilution, HisDetector Nickel-HRP, KPL Inc, USA). Binding was detected using chemiluminescent substrate. His tagged recombinant proteins in Lanes: A - LdCK1.4; B - LdCK1.4Δ^1–90^; C - LdCK1.4Δ^411–566^, and D - LdCK1.4Δ^1–90,411–566^.

Full-length (*Ldck1.4*) and three deletion constructs (Figure 3A) were cloned into pET28a, and the polypeptides expressed in *E. coli* by induction with IPTG and arabinose. Western blotting analysis detected major bands representing each His-tagged CK1.4 polypeptide at appropriate molecular weight in lysates from the induced bacteria (Figure 3B Lanes A+ to D+). The predicted molecular mass of each recombinant polypeptide is: full-length LdCK1.4, 62 kDa; LdCK1.4Δ^1–90^, 51 kDa; LdCK1.4^411–566^, 45 kDa; and LdCk1.4^1–90, 411–566^, 36 kDa. Some smaller proteolytic degradation products were also noted. No His-tagged CK1.4 polypeptides were detected in lysates of non-induced bacteria (Figure 3B, lanes A- to D -).

The protein kinase activity of the full-length LdCK1.4 and truncated recombinant polypeptides was tested using the induced bacterial lysates on hydrolyzed casein as substrate, and compared to non-induced bacterial lysates (negative control). Casein kinase activity (46,600 cpm ±2800 s.e.), approximately 2.7-fold higher (t-test, p = 0.012) than the negative control (17,100 cpm ±1700 s.e.), was only detected in bacteria expressing the full-length enzyme. No increase in protein kinase activity was observed when induced bacterial lysates expressing the three truncated polypeptides were examined (data not shown), and further studies characterizing recombinant LdCK1.4 activity are planned using a kinase-dead recombinant enzyme as a negative control. Therefore, only the full-length casein kinase gene was used to transfect parasites.

### 3. LdCK1.4-FLAG Expression in Stably Transfected Promastigotes

**Figure 4:**
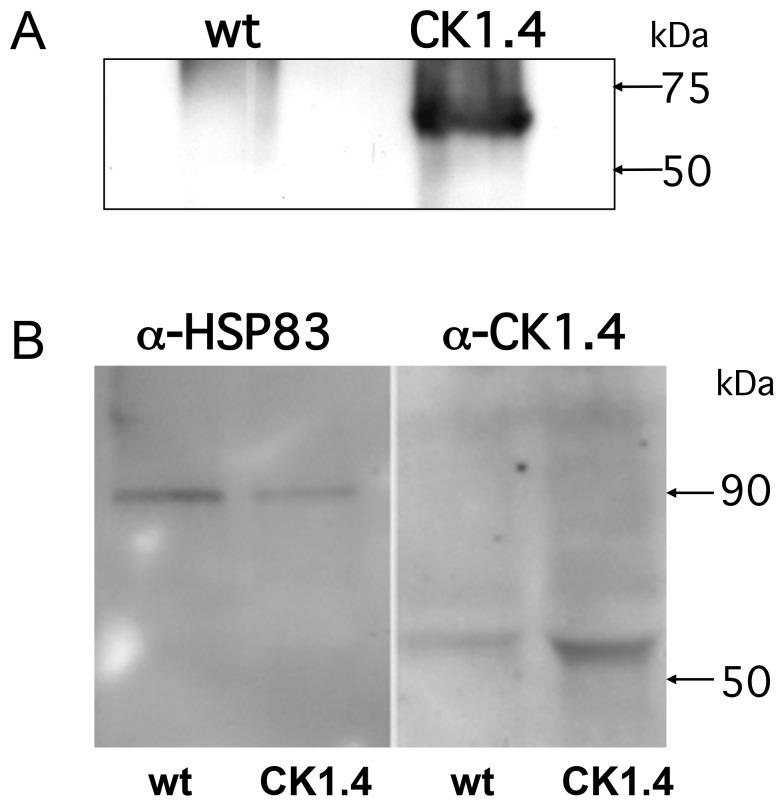
Expression of CK1.4-FLAG by mutant *L. donovani* promastigotes (Ld:CK1.4-FLAG). SDS-PAGE and Western blot analysis of total cellular lysates (10^7^ promastigotes/lane) from wild type (wt) and Ld:CK1.4-FLAG (CK1.4) promastigotes using Panel A: anti-FLAG M2 monoclonal antibody (1/1000 dilution, Sigma - Aldrich Chemical Co.) or Panel B: rabbit polyclonal anti-HSP83 (1/5000 dilution) or anti-CK1.4 (1/7500 dilution).

In order to examine the effect of CK1.4 on the parasite growth and morphology, and determine whether or not this protein kinase is secreted, carboxy-terminus labeled LdCK1.4-FLAG was stably over expressed in *L. donovani*. Promastigotes were transfected with the leishmanial expression vector (pSSU:LdCK1.4-FLAG), and mutant parasites selected by growth in hygromycin. Western blot analysis of total parasite lysates (Figure 4A) showed that FLAG-tagged rLdCK1.4 is expressed by the mutant promastigotes (Ld:CK1.4), but not wild-type parasites (Ld:wt). A strong band at ∼62 kDa is detected in lysates of the mutant parasites using anti-FLAG antibodies, but not seen with the Ld:wt parasites. In order to examine the level of CK1.4 over expression, lysates from Ld:CK1.4-FLAG mutants and Ld:wt parasites were examined by Western blotting using rabbit polyclonal anti-HSP83 and –CK1.4 serum (Figure 4B). Densitometry of the blots, and normalization of the amount of material in each lane based on reaction with anti-HSP83 antibodies showed that CK1.4 expression was ∼6.4-fold higher in the LdCK1.4-FLAG mutants than the Ld:wt parasites.

**Figure 5:**
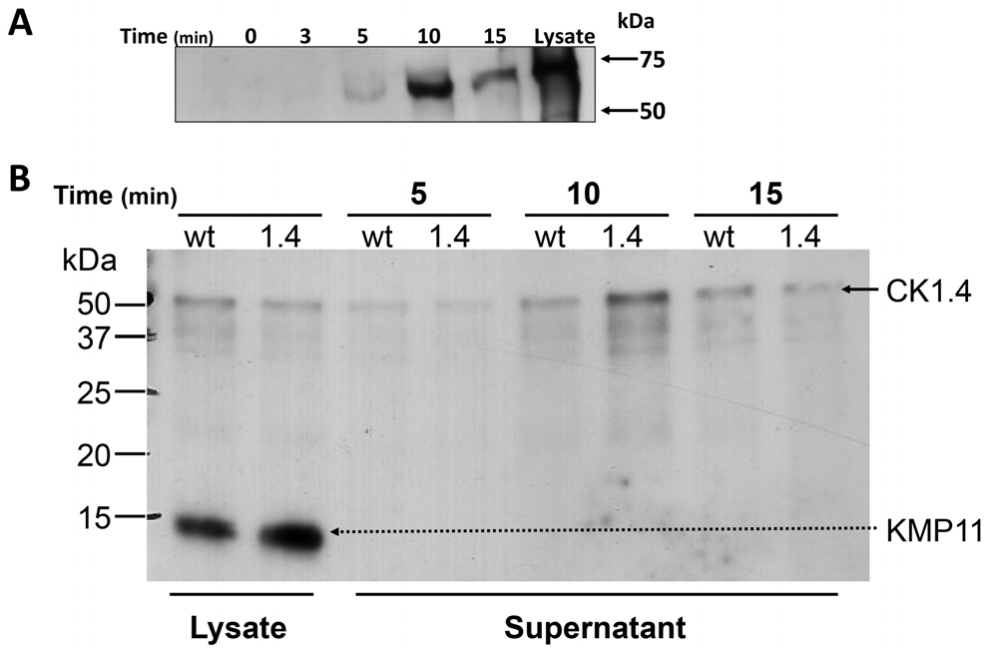
Time course of CK1.4 release by *Leishmania donovani* promastigotes. Cell-free supernatants were prepared from Ld:wt and/or Ld:CK1.4-FLAG parasites at different times (min) post-induction with ionomycin (5 µM)/EGTA (1 mM), and examined by SDS-PAGE - Western blotting. Panel A: Membranes were incubated with anti-FLAG M2 monoclonal antibody (1/1000 dilution, Sigma –Aldrich Chemical Co); Lysate is a positive control prepared from 10^7^ Ld:CK1.4-FLAG promastigotes collected at t = 0. Panel B: Membranes probed simultaneously with rabbit polyclonal anti-CK1.4 (1/7500 dilution) and anti-KMP 11 (1/2000 dilution) antibodies. Lanes labeled Lysate were prepared from either 10^7^ Ld:wt or Ld:CK1.4 promastigotes collected at t = 0 min. Antibody binding was detected by incubation of the membrane with a chemiluminescent substrate and exposure to X-ray film.

Release of LdCK1.4-FLAG into cell-free supernatants by the mutant promastigotes over time was followed for 15 min (Figure 5). Induced release of casein kinase was initiated by addition of promastigotes to buffer A containing ionomycin and EGTA. Lowering intracellular [Ca^+2^] induces protein kinase release, similar to that found when exogenous protein kinase substrates such as phosvitin or casein are added to the parasites [10]. At each time point, aliquots containing equal numbers of parasites were removed, cell free supernatants prepared, and CK1.4 release examined by Western blotting with anti-FLAG or rabbit anti-CK1.4 antibodies. Parasite viability, 97%, was unchanged over the course of the experiment. The initial time point (t = 0) was obtained by centrifuging the parasites immediately following ionomycin induction. Whole cell lysates prepared from LdCK1.4-FLAG mutant promastigotes were used as positive controls, and a rabbit anti-KMP11 antibody was used to monitor protein release by dead or dying cells.

The presence of LdCK1.4-FLAG in the cell-free supernatant was not observed by Western blotting with anti-FLAG antibodies (Figure 5A) at t = 0 min, and only a very weak reaction was observed at t = 3 min (Δintensity: density band / density background = 1.32). However, by 5 min post-induction the presence of LdCK1.4-FLAG in the cell-free supernatant was readily apparent (Δintensity = 3.26). The level of secreted enzyme peaked at 10 min post-induction (Δintensity = 18.0), and then decreased slightly at 15 min (Δintensity = 10.7). A strong reaction by the anti-FLAG antibodies with CK1.4-FLAG in the promastigote lysates was also observed (Lysate). The kinetics of CK1.4-FLAG release is very similar to that previously noted for a CK1-like activity released by *L. major* promastigotes [10], which also peaked at 10 min.

A similar pattern of CK1.4 release from the Ld:CK1.4-FLAG mutant promastigotes was observed when polyclonal anti-CK1.4 was used instead of anti-FLAG antibodies. This experiment was repeated three times (Figure S2), and results from a typical experiment are shown in Figure 5B. No reaction with the supernatants collected from Ld:CK1.4-FLAG or Ld:wt parasites was observed at t = 0 or 3 min (Figure S2, and data not shown), however a weak band was observed with Ld:CK1.4-FLAG promastigotes (Figure 5B, Supernatant, lanes –1.4) at 5 min (Δintensity: density band / density background = 2.0), peaking at 10 min (Δintensity = 12.1), and then decreasing at 15 min (Δintensity = 5.47). Release of CK1.4 by Ld:wt parasites (Figure 5B, Supernatant, lanes − wt) was also observed at 5 min (Δintensity = 1.7), and increased with time. However, the kinetics of CK1.4 release by the wt parasites appeared to be somewhat different than the mutant parasites (Figure S2). While the level of secreted CK1.4 at 5 min for both wt and mutant parasites was similar (Δintensity = 1.7 wt versus 2.0 mutant), the intensity observed at 10 min for the wt promastigotes (Δintensity = 5.6) was only 46% that seen for the mutants. Interestingly, unlike the mutant parasites, the level of CK1.4 in the wt supernatants did not decrease at 15 min (Δintensity = 6.8), but was similar to that seen at 10 min.

The membrane was also probed with polyclonal anti-KMP 11 antibodies. KMP 11 is present in the cytoplasm and flagellar pocket, as well as on the surface and flagella of the parasite [35], [36]. KMP 11 was only detected in the whole parasites lysates (Figure 5B, Lysate), and not in any of the cell-free supernatants examined (Figure 5B, Supernatant and data not shown). This finding further indicates that the presence of CK1.4 in the cell-free supernatants is not due to cell death.

### 4. Localization of CK1.4 in *L. donovani* Promastigotes

**Figure 6:**
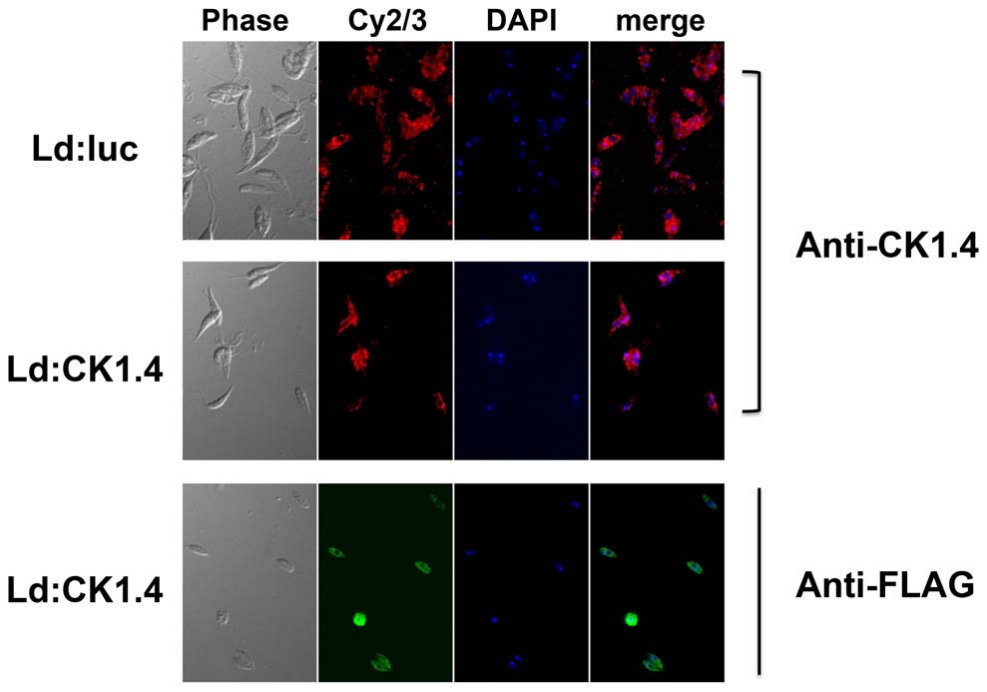
Localization of CK1.4 in *Leishmania donovani* promastigotes by immunefluorescent staining with anti-FLAG and anti-CK1.4 antibodies. Logarithmic stage Ld:CK1.4-FLAG (Panels Ld:CK1.4) and Ld:LUC (Panels Ld:luc) promastigotes were fixed in 4% paraformaldehyde, centrifuged onto poly-L-lysine coated slides, and permeabilized with ice cold methanol. The slides were incubated with anti-FLAG M2 monoclonal antibodies (1/500 dilution), rabbit anti-CK1.4 polyclonal antibodies (1/7500 dilution), or buffer alone. Staining was carried out by incubating slides in appropriate secondary antibody, either Cy2-goat anti-mouse IgG or Cy3-goat anti-rabbit IgG (Panel Cy2/3; 1/200 dilution), and mounted in Fluoroshield with DAPI (Panel DAPI). The immunofluorescence examined using an Apochromat oil immersion objective (100×magnification) on an Olympus IX71S8F microscope.

Immunofluorescence was utilized to localized and compare the expression of the FLAG labeled and native protein kinase in Ld:CK1.4-FLAG and Ld:LUC promastigotes (Figure 6, Panels Ld:CK1.4 and Ld:luc, respectively). Actively dividing promastigotes were fixed with paraformaldehyde and incubated with either antibodies to the FLAG epitope, to recombinant CK1.4, or buffer alone (Figure 6, and data not shown). The fluorescence was examined after incubation with either Cy2- or Cy3 labeled secondary antibodies. The distribution of native CK1.4 in either Ld:CK1.4-FLAG or Ld:LUC (red, Panels Cy2/3) parasites appears to be the same. In both parasites the fluorescence is intracellular, and present both as weak diffuse staining throughout the cytoplasm, as well as stronger punctate pattern adjacent to the nucleus and/or kinetoplast, but not co-localizing with the nuclear DAPI staining (red, Panels merged). Intensity of CK1.4 staining varies markedly between individual parasites, perhaps depending on the cell cycle. When anti-FLAG antibodies are used, the fluorescence pattern observed with Ld:CK1.4-FLAG promastigotes (green, Panel Cy2/3) is essentially identical to that observed using anti-CK1.4 antibodies (red, Panels Cy2/3). Similar to the anti-CK1.4 antibodies, staining of the Ld:CK1.4-FLAG parasites with anti-FLAG antibodies gives a weak diffuse fluorescence in the cytoplasm, as well as strong punctate staining adjacent to the nucleus and/or kinetoplast. This fluorescence does not co-localize with the nuclear DAPI staining (green, Panel merged). No fluorescence was observed when anti-FLAG antibodies were incubated with Ld:LUC promastigotes or when the primary antibodies were omitted, negative controls (data not shown).

### 5. Effect of CK1.4 Over Expression on Promastigote Growth and Metacyclogenesis

**Figure 7:**
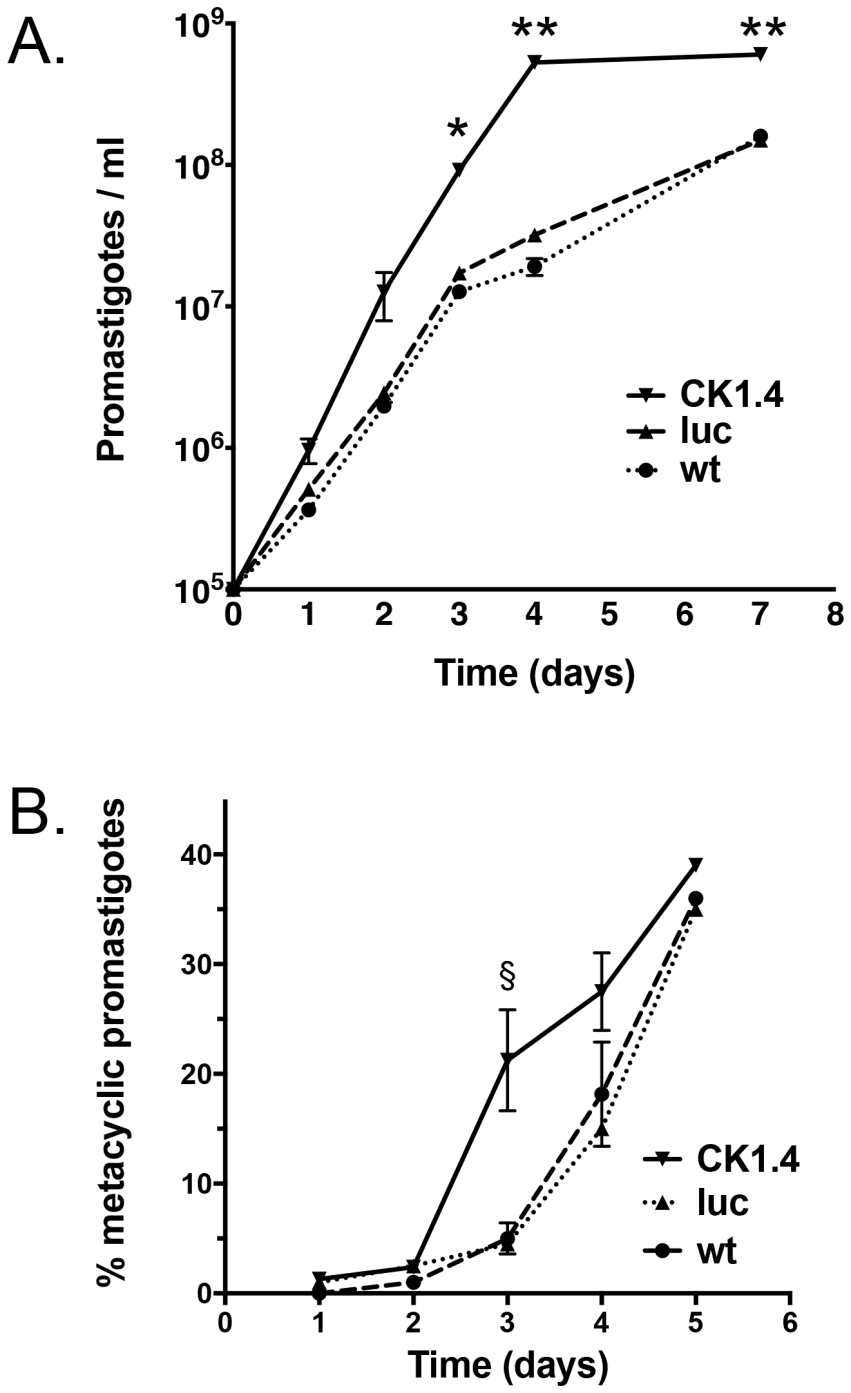
Comparison of wild-type and mutant promastigote growth and metacyclogenesis in culture. Promastigotes, Ld:wild type (wt), Ld:LUC (luc) and Ld:CK1.4-FLAG (CK1.4) expressing mutants, were diluted (10^5^ cells/ml) at day 0 in complete culture media. Panel A. Parasite growth was monitored daily by counting live cells in a haemocytometer. All experiments were performed in triplicates. *, P<0.01 and **, P<0.001. Panel B. Analysis of parasite differentiation into metacyclic promastigotes over time in culture. Forward (FSC) and side scatter (SSC) properties of cultures, wt, luc and CK1.4, were examined daily by flow cytometry. Gating for metacyclic and procyclic promastigotes, Figure S1, was determined by comparing parasite distribution before and after separation of these populations on Ficoll gradients using stationary phase parasites [32]. §, P = 0.04.

The growth of Ld:wt, Ld:LUC and Ld:CK1.4-FLAG promastigotes in culture were followed over seven days by either counting in a Neubauer haemocytometer or using the fluorescent viability reagent, alamarBlue. Promastigotes were seeded at identical densities (5×10^5^ cells/ml) on day 0, and the cell concentration determined daily. By both assays the Ld:CK1.4-FLAG promastigotes were shown to grow faster and to higher cell densities than the Ld:wt and Ld:LUC parasites (2-way ANOVA P<0.001). Results obtained by counting are shown in Figure 7A. No significance difference between the three cultures was noted at early time points, 24 and 48 hrs after passage, though the Ld:CK1-FLAG parasites already appear to have reached a higher cell density than the control cultures. However by day 3, the density of the CK1.4-FLAG promastigotes was significantly higher (P<0.01), 9.2×10^7^ cells/ml, than either of the control cultures (wt = 1.3×10^7^/ml; LUC = 1.7×10^7^/ml), and remained significantly higher (P<0.001) on day 4 and day 7. Ld:CK1.4-FLAG promastigotes reach a density of 6.1×10^8^ cells/ml on day 7 compared to 1.6 and 1.5×10^8^ cells/ml for the Ld: wt and Ld:LUC parasites, respectively. No significance difference between the Ld:wt and Ld:LUC promastigote growth was noted.

Similar results were observed when cell growth was monitored daily using the viability indicator alamarBlue, where fluorescence (λ_ex_ = 544 nm; λ_em_ = 590 nm) is proportional to cell density (data not shown). No significant difference in fluorescence between the parasite cultures was noted 24 hrs after passage. However, at 48 hrs fluorescence for parasites over expressing CK1.4 was ∼50% higher than the controls. All the cultures entered stationary phase by day 4, and there was little additional increase in fluorescence on day 6. On both day 4 and 6 the fluorescence was significantly higher, 76 and 81% (P<0.05), for the Ld:CK1.4-FLAG promastigotes than the control parasites indicating that former cultures reached a higher cell density.

In order to examine whether CK1.4 over expression also effects parasite differentiation in culture, we followed mutant and wild-type promastigote metacyclogenesis daily by measuring the forward-angle scatter (FSC) and side-angle scatter (SSC) parameters using flow cytometry [31]. Gating of the procyclic and metacyclic promastigote populations (Figure S1) was determined using pure parasite populations following separation by centrifugation on a Ficoll step gradient [32]. Experiments were repeated twice. The percentage of metacyclic promastigotes present in all three cultures was very low, 0–2.5%, on days 1 and 2 after passage (Figure 7B). However, by day 3 a significant percentage of metacyclic promastigotes was observed in the Ld:CK1.4-FLAG culture, 21.5%. This was approximately four times greater than that observed for the Ld:wt or Ld:LUC parasites, 5.0% or 4.0%, at this time (t-test, P = 0.04). By day 4 the Ld:wt and Ld:LUC parasites began to catch up to the Ld:CK1.4-FLAG parasites with the metacyclic population in the control cultures now comprising 18.2% and 15%, respectively, of the promastigotes versus 27.5% for the Ld:CK1.4-FLAG promastigotes. After five days, when both parasite populations are in the stationary phase of growth, and essentially stopped dividing, the percentage of metacyclic promastigotes for the populations was similar (36% Ld:wt; 35% Ld:LUC and 39% Ld:CK1.4-FLAG). Interestingly, while the final percentage of metacyclic parasites is similar for all the parasite populations, the Ld:CK1.4 mutants differentiate into the virulent stage of the parasite earlier than the wild type parasites.

### 6. Infection of Macrophages by Wild Type and Mutant Parasites

In order to see whether CK1.4 over expression also affects parasite virulence and survival, the ability of Ld:CK1.4-FLAG and wild type promastigotes to infect BALB/c mouse peritoneal macrophages was compared using day 5 stationary phase parasites that contain similar percentages of metacyclic promastigotes. The percentage of infected macrophages, and number of amastigotes per infected macrophage was determined 72 hrs post-infection. The experiment was repeated three times. Over expression of CK1.4 significantly increased, ∼4.5– fold (p<0.0005), the percentage of infected macrophages (28.63±2.60%) compared to the wild type parasites (6.32±2.08%). Only a small, but significant difference in the number of mutant and wild type parasites per infected macrophages was noted, 3.45 versus 2.04 amastigotes/macrophage (p<0.004), respectively.

## Discussion

Casein kinase 1 is involved in the regulation of biological processes including cell growth, transport, metabolism and apoptosis [16], [17]. As this protein kinase does not contain a regulatory subunit, subcellular localization and phosphorylation is thought to be important in controlling CK1 interaction with cell substrates and regulating the function of this protein kinase. In yeast, CK1 isoforms are targeted to either the cell membrane (Yck1 and Yck2) or the nucleus (HRR25), and have been shown to be essential for cell growth [37]. However, no nuclear localization signal or other motif targeting LdCK1.4 to a specific subcellular compartment was identified.

Similar to other eukaryotic cells, *Leishmania* and Trypanosomes have several CK1 isoforms [18]. While most leishmanial CK1 isoforms have orthologs in *T. cruzi* or *T. brucei*, if not all three species, the gene coding for CK1.4 is unique to *Leishmania*, and has not been identified in any other kinetoplastids examined to date [19]. Phylogenetic analysis of leishmanial CK1 indicate that LCK1.1 and LCK1.2 are evolutionary more similar to typical mammalian and parasite casein kinases, such as human CK1-α, -δ and -ε, *T. gondii* CK1-α and -β, and *Plasmodium*, while *Leishmania* CK1.4, together with isoform 3 (LdCK1.3, LinJ.04.1230), forms a distinct evolutionarily group removed from the other CK1s.

To date limited work on the role of casein kinases in *Leishmania* has been undertaken. These protein kinases are interesting because they are thought to be potential drug targets [38], [39]. CK1 inhibitors, while not necessarily specific for isoform 2, blocked *L. major* promastigote and *T. brucei* trypomastigote growth at µM concentrations [21], [40]. No attempts to our knowledge have been made to produce LCK1.2 null mutants. However double knockout of the *T. brucei ck1.2* gene (Tb927.5.800) was unsuccessful suggesting that this gene is essential [20], and knock-down of *Tbck1.2* expression using RNAi caused morphological changes, and was ultimately lethal to the parasite [20]. Interestingly, null mutants for another isoform TbCK1.1 (Tb927.5.790), 72% identical to TbCK1.2, were not lethal, and caused no phenotypic changes suggesting CK isoforms perform different functions in these parasites [20].

Previous studies have shown that CK1 and CK2 are released from promastigotes either constitutively and/or following induction, and that secretion is modulated by the external environmental pH [10], [11]. Mechanisms of protein secretion in *Leishmania* are not well understood, but are thought to take place via a classical amino terminal secretion signal pathway, as well as membrane blebbing or microvesicle release from the flagella pocket or cell membrane [41], [42]. Analysis by mass spectrometry of culture medium or exosomes collected from *L. donovani* promastigotes after longer incubations, 4–6 hrs or 24 hrs respectively, identified numerous proteins, including KMP-11, LdCK1.2 and other protein kinases [8]. Further analysis of parasite exosomes present in 4–6 hrs old culture medium showed that LdCK1.2 is among the 329 proteins secreted in microvesicles from *Leishmania,* and that exosome secretion is affected by both pH and temperature [12], [43]. LdCK1.4 has not been reported among proteins found in the parasite exosome or secretome, suggesting either LCK1.4 is not present in exosomes, does not accumulate over time in spent culture medium or is degraded by the parasite. The absence of KMP-11, a cell membrane and cytoplasmic protein, in the cell-free supernatants (Figure 5) following short periods of incubation indicates that LdCK1.4 release by the promastigotes is not due parasite lysis or release of exosomes. Immunofluorescence indicates that LdCK1.4 is an intracellular protein and not present on the promastigote surface membrane. However, we have shown that this protein kinase is released from promastigotes. Interestingly, the short-term kinetics of LdCK1.4 release following induction shows that the amount of protein in the cell-free supernatants peaks at 10 min and then decreases. This suggests that phosphorylation of host or parasite proteins by released CK1.4 may act in a temporal fashion, and that its release may be induced by specific environmental or cellular cues. Interestingly, Liu et al. (2009) showed that a leishmanial ortholog of human CK1-α could regulate IFNAR1 stability and type I interferon signaling in macrophages.

Both CK1.2 and CK1.4 are expressed in all stages of the *Leishmania* life cycle, however expression of CK1.2 decreases during promastigote differentiation to amastigotes, and is lower in the intracellular stage of the parasite. On the other hand, expression of CK1.4 increases rapidly during differentiation from promastigotes to amastigotes, and is 4.2-fold higher in the intracellular stage of the parasite [26], [44], [45] (Beverley et al., and Zilberstein and Myler et al., unpublished data - http://tritrypdb.org/tritrypdb/). The increase in LdCK1.4 expression and higher expression in amastigotes suggests that this protein kinase may play a role in parasite differentiation and survival in the intracellular host environment. Indeed, over expression of LdCK1.4 in promastigotes caused marked changes in parasite phenotype causing them to differentiate earlier into the virulent metacyclic form, and reach higher cell densities in culture than wild-type parasites. Mutant LdCK1.4 promastigotes caused significantly higher macrophages infections than wild type parasites. We are performing additional experiments to investigate this phenotype in detail including comparison with a cell line expressing a kinase-dead mutant of the enzyme. Interestingly, in silico analysis of the *L. major* interactome predicts that LCK1.4 is probably essential for parasite survival, and maybe a putative drug target [39]. Further characterization of pure CK1.4, its mechanism of secretion, and its role in parasite survival should allow us to establish the potential of this unique leishmanial protein kinase as a putative drug target.

## Supporting Information

Figure S1 **Separation on Ficoll gradients, and analysis of** ***Leishmania donovani*** **metacyclic promastigotes by flow cytometry.** Metacyclic promastigotes were purified from stationary phase promastigotes essentially as previously described for *L. major* and *L. chagasi* [32], [33] and used to establish the correct gating for procyclic and metacyclic promastigote populations. Parasites were washed and suspended in RPMI-1640 (10^9^ cells in 2 ml). The cells were carefully layered on top of a step gradient (10% Ficoll : 40% Ficoll - 2 ml each concentration) and centrifuged at room temperature (10 min, 360×g, no brake). Parasites were collected from each band, and three fractions: 1) before separation, 2) 0%:10% interface and 3) 10%:40% interface analyzed by flow cytometry. For analysis the cells were stained with propidium iodide (0.1 mg/ml) for 5 min, washed by centrifugation with PBS containing 2% FCS and 0.01% sodium azide, and finally suspended in this buffer. Forward and side scatter parameters were collected with a flow cytometer (CyAn™ ADP Dako, Carpinteria CA, USA) and analyzed using Summitv4.3 software.
(TIF)

Figure S2 **Analysis of CK1.4 release from** ***Leishmania donovani*** **promastigotes.** Cell-free supernatants were prepared from Ld:wt (wt) and/or Ld:CK1.4-FLAG (CK1.4) parasites at different times (min) post-induction with ionomycin (5 µM)/EGTA (1 mM), and examined by SDS-PAGE - Western blotting. CK1.4 release was analyzed by incubation with rabbit anti-CK1.4 polyclonal antibody followed by Protein A – HRP. Binding was detected by reaction with chemiluminescent substrate and exposure to X-ray film. The relative band intensity at each time point compared to background (Δ Intensity ± s.e.) for all the experiments was analyzed by densitometric analysis with NIH Image program (developed at the U.S. National Institutes of Health and available on the Internet at http://rsb.info.hih.gov/hih-image), and plotted.
(TIF)
