# Supplementary figures and images for: Identification of a Secreted Casein Kinase 1 in Leishmania donovani: Effect of Protein over Expression on Parasite Growth and Virulence
Source: PLoS One. 2013 Nov 15;8(11):e79287. doi: 10.1371/journal.pone.0079287 (PMC3829951; doi:10.1371/journal.pone.0079287)

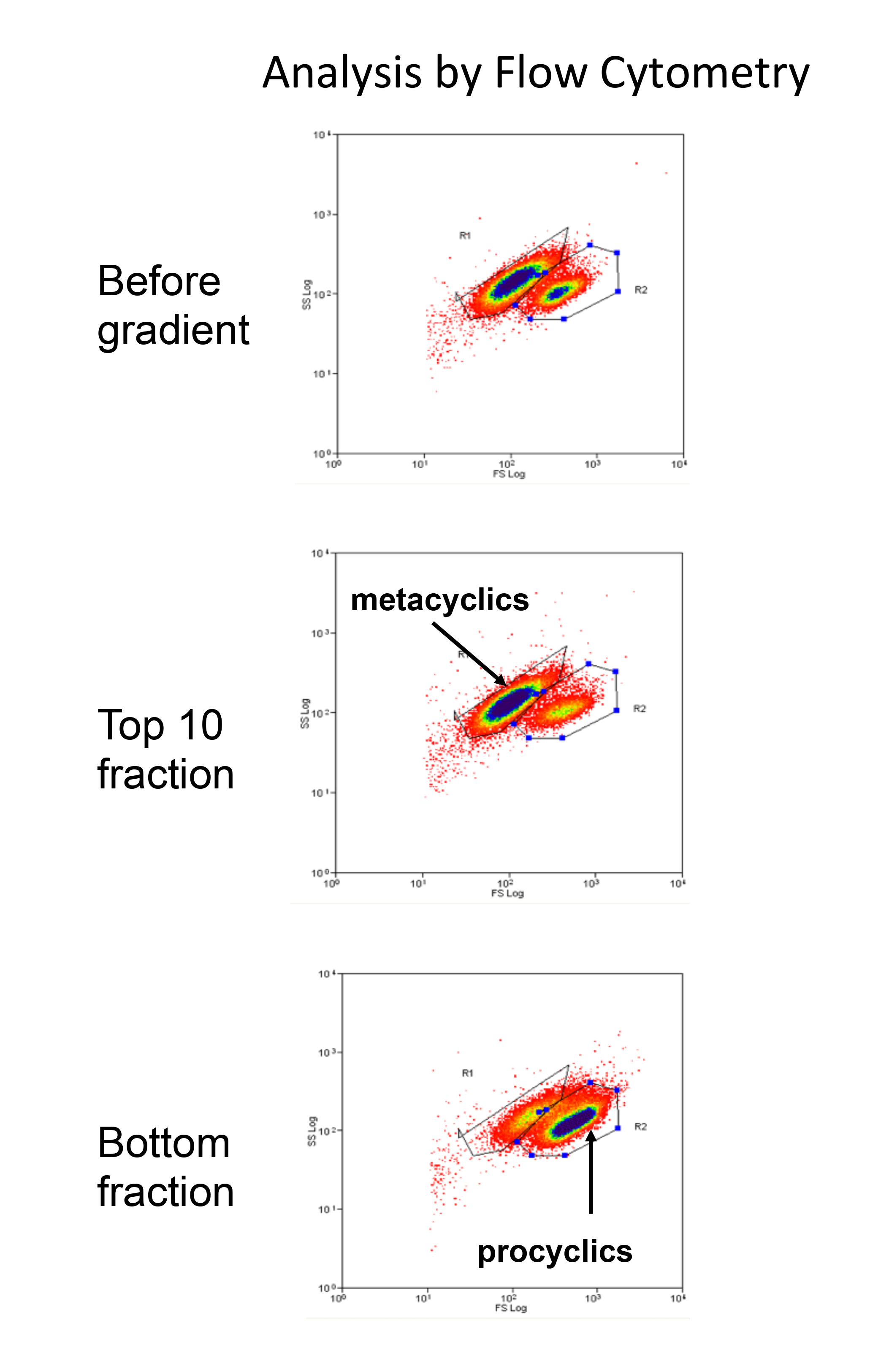

Supplement: Figure S1 — Separation on Ficoll gradients, and analysis of Leishmania donovani metacyclic promastigotes by flow cytometry. Metacyclic promastigotes were purified from stationary phase promastigotes essentially as previously described for L. major and L. chagasi [32], [33] and used to establish the correct gating for procyclic and metacyclic promastigote populations. Parasites were washed and suspended in RPMI-1640 (109 cells in 2 ml). The cells were carefully layered on top of a step gradient (10% Ficoll : 40% Ficoll - 2 ml each concentration) and centrifuged at room temperature (10 min, 360×g, no brake). Parasites were collected from each band, and three fractions: 1) before separation, 2) 0%:10% interface and 3) 10%:40% interface analyzed by flow cytometry. For analysis the cells were stained with propidium iodide (0.1 mg/ml) for 5 min, washed by centrifugation with PBS containing 2% FCS and 0.01% sodium azide, and finally suspended in this buffer. Forward and side scatter parameters were collected with a flow cytometer (CyAn™ ADP Dako, Carpinteria CA, USA) and analyzed using Summitv4.3 software. (TIF) [file pone.0079287.s001.tif]

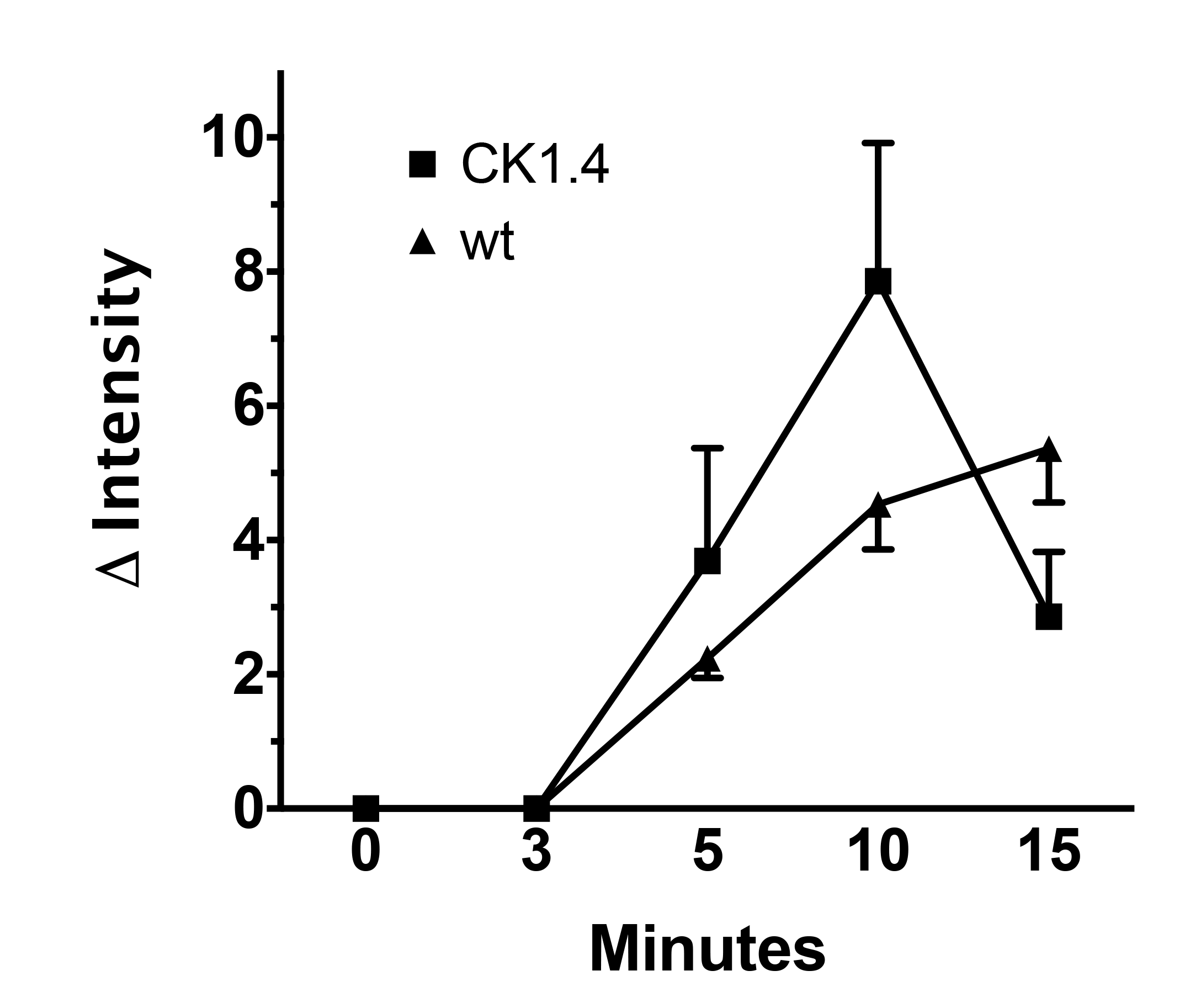

Supplement: Figure S2 — Analysis of CK1.4 release from Leishmania donovani promastigotes. Cell-free supernatants were prepared from Ld:wt (wt) and/or Ld:CK1.4-FLAG (CK1.4) parasites at different times (min) post-induction with ionomycin (5 µM)/EGTA (1 mM), and examined by SDS-PAGE - Western blotting. CK1.4 release was analyzed by incubation with rabbit anti-CK1.4 polyclonal antibody followed by Protein A – HRP. Binding was detected by reaction with chemiluminescent substrate and exposure to X-ray film. The relative band intensity at each time point compared to background (Δ Intensity ± s.e.) for all the experiments was analyzed by densitometric analysis with NIH Image program (developed at the U.S. National Institutes of Health and available on the Internet at http://rsb.info.hih.gov/hih-image), and plotted. (TIF) [file pone.0079287.s002.tif]
